# Outcomes of Revision surgery for surgically treated insertional Achilles tendinopathy

**DOI:** 10.1007/s00402-024-05693-9

**Published:** 2024-12-16

**Authors:** Hubert Hörterer, Sonia Oppelt, Kathrin Pfahl, Norbert Harrasser, Wolfgang Böcker, Hans Polzer, Markus Walther, Sebastian Felix Baumbach

**Affiliations:** 1 Center for Foot and Ankle Surgery, Schön Klinik München Harlaching, Munich, Germany; 2 Department of Orthopaedics and Trauma Surgery, Musculoskeletal University Center, Munich (MUM), University Hospital, LMU Munich, Munich, Germany; 3 Clinic of Orthopaedics, Klinikum Rechts Der Isar, Technical University Munich, Munich, Germany; 4 Department of Orthopedics and Orthopedic Surgery, Julius-Maximilians-University, Würzburg, Germany

**Keywords:** Revision surgery, Insertional achilles tendinopathy, Haglund Deformity, Haglund Syndrome

## Abstract

**Introduction:**

There is a clear roadmap for the treatment of primary insertional Achilles tendinopathy (IAT), but data on the outcome of revision surgery is missing. The current study aimed to analyze the outcome following revision surgery for surgically failed IAT.

**Material and methods:**

Included were patients with IAT revision surgery at a single reference center (01/2010–10/2016) and a follow-up of at least 12 months. Revision surgery was performed, whenever possible, through a midline incision transachillary approach (MITA) with debridement of all pathologies present. The patient-rated outcome was assessed per the FFI (preoperative, final follow-up) and VISA-A-G (final follow-up). The aim was to evaluate the patient rated outcome following revision surgery for recurrent IAT.

**Results:**

Out of 24 eligible patients, 19 (79%) were included in the final follow-up. The mean follow-up duration was 4.6 ± 2.2 years. The FFI Overall improved from preoperatively 68 ± 19 to 14 ± 17 points (< 0.001) at the final follow-up. The final VISA-A-G was 71 ± 28 points. 39%/36% (FFI/VISA-A-G) of patients reached patient-rated outcome scores comparable to a healthy reference population. No factors could be identified to influence the outcome significantly.

**Conclusion:**

IAT revision surgery results in an improvement of the patients’ symptoms, but only one-third of the patients recover fully.

## Introduction

Insertional Achilles tendinopathy (IAT) remains a challenge for every orthopedic physician. The published lifetime risk of achilles tendinopathy ranges between 5 to 18%, rising up to 50% among runners [1, 2]. There appears to be a general consensus on the treatment algorithm for primary IAT. Conservative treatment options are the first-line approach, including extracorporeal shockwave therapy [3–5] and eccentric exercises [4, 5] over three to six months. Still, in almost 30% of patients, conservative treatment for IAT fails, and surgical treatment options must be discussed [6].

Most authors favor the debridement of all pathologies present at the insertion of the Achilles tendon by open surgery [7]. Pain causing pathologies include the retrocalcaneal and superficial bursitis, degenerative changes in the Achilles tendon with intratendinous calcifications or a dorsal heel spur in the Achilles tendon [8]. The most commonly applied surgical procedure is a midline incision, trans-achillary approach with debridement of all pathologies present [7, 9]. This treatment approach will result in significant pain relief in 90% of the patients. Still, about half of the patients suffer from residual impairment [9–13]. Alternative surgical strategies include endoscopic techniques in favor of wound healing disorders as well as derotational osteotomies of the calcaneus to reduce the retrocalcaneal pressure and to optimize the mechanics of the insertion of the Achilles tendon (AT) [14, 15]. The latter surgical procedure is also increasingly performed percutaneously and has yielded promising results in published case series [16, 17]. Although precise data are lacking, recent findings suggest that the recurrence rate for primary surgical IAT treatment using a midline incision and transachillary approach (MITA) is between 7 and 17%. [18].

As well-defined as the treatment algorithm for primary IAT is, it is unclear how to treat recurrence cases as literature on revision surgery is little to non-existent [19]. The first line of treatment in recurrence will again be a conservative approach. If unsuccessful, surgeons will face the situation of discussing revision surgery with their patients. Still, we can not present valid figures to our patients, as data on the outcome of revision surgery are missing. Therefore, the current study aimed to analyze the outcome following revision surgery for surgically failed IAT.

## Material and methods

The herein-presented study is a retrospective case-series utilizing the authors’ in-house database. The study has been approved by the local ethics committee (#17–804).

### Patient selection

Eligible were adult patients (age ≥ 18 years) who had revision IAT surgery due to a failed initial surgical treatment for IAT between 01/2010 and 10/2016. The revision surgery must have been conducted because of worsening of isolated, unilateral persistent complaints at the insertion of the Achilles tendon for more than 12 months after surgery, with failed nonoperative treatment for more than 6 months. Finally, patients must have been available for a current follow-up. Patients with bilateral IAT, mid-portion Achilles tendinopathy, or any other concomitant diseases associated with tendinopathy or affecting the outcome, for example, rheumatism, or other foot and ankle disorders, were excluded from this study. The study also did not include patients who were pregnant.

### Surgical treatment strategy

Overall, the revision surgery followed the same principles as the primary surgery for IAT, outlined previously [9, 20]. Whenever possible, a midline incision transachillary approach (MITA) was used to address all pathologies possibly responsible for the patients’ persistent complaints. If needed, the surgical approach was adapted according to the preexisting approach and extended proximally or distally as needed. If necessary, the Achilles tendon was freed from any scar tissue and, whenever possible, a separate layer, similar in function to the peritendineum, was prepared. Following the transachillary split, the tendon was inspected for any possible degenerative changes or calcifications. If present, these were debrided. The tendon was detached from the calcaneal tuberosity as needed to remove any bony prominences at the insertion of the Achilles tendon. Any scar tissue within the retroachillary space was debrided.

The postoperative protocol was identical to that of a primary IAT surgery [9, 20]. Patients were instructed to perform ten kilogram (kg) of partial weight bearing with crutches for two weeks and then progress to pain-dependent full weight bearing per their individual capacities. The objective for the patient was to achieve ambulation without the use of crutches within eight weeks. The walker was placed in a neutral position for six weeks if the Achilles tendon was less than 50% detached from its insertion point. If the Achilles tendon was detached more than 50% from its insertion, a reattachment by suture anchor was performed. Therefore, patients were advised to wear a walker in 120° of plantarflexion for four weeks, followed by 105° and 90° for another two weeks each. This protocol was the same following a FHL transfer.

### Data assessment and analysis

All eligible patients were contacted for a current follow-up. The present follow-up included the following patient-reported outcome measures (PROMs): the foot function index (FFI) [21] and the Victorian Institute of Sport Assessment—Achilles (VISA-A-G) questionnaire [22]. The functional foot index (FFI) serves as our institution’s primary quality assessment tool and is administered voluntarily. It is automatically assessed preoperatively and mailed to each patient for completion at their 12-month follow-up. Due to this process, the FFI serves as our primary outcome parameter. Based on the data available, the target range for a healthy average population was chosen for the FFI as 0–5 points and for the VISA-A-G ≥ 90 points.[22–25]. Next to the current follow-up, the same demographics, medical history, and surgical details were assessed as outlined previously [18]. Analyzed were the final follow-up PROM scores, the FFI improvement preoperatively to last follow-up and possible factors affecting the outcome of revision surgery. To identify possible factors affecting the postoperative patient-rated outcome, the patients were divided into two groups per the FFI. A good outcome was defined as a FFI Overall score in the range of the average population (FFI ≤ 5 points). The outcome of the remaining patients was considered impaired. Complications were categorized into major and minor complications [9]. In this context, minor complications were defined as complications that did not necessitate further surgery. This included, for example, instances of minor surgical site infections, defined as any delayed wound healing, for which the recommended treatment was superficial wound debridement and oral antibiotics if necessary. In contrast, major complications were defined as any complications that necessitated surgical intervention or presented a potentially life-threatening condition.

## Statistics

The FFI scores revealed a normal distribution per the Shapiro–Wilk Test (*p* = 0.172 - *p* = 0.069). Therefore, parametric statistics were applied, and all values are given as mean ± SD if not stated differently. The statistics used were standard descriptives, paired and unpaired, two-tailed students t-tests, and the chi-squared tests (Fischer exact test), where appropriate. The significance level for the FFI, with its 3 subscales, was adapted per a Bonferroni alpha-level correction to p < 0.017. For all further analysis, a Bonferroni alpha-level correction set the significance level to p < 0.004.

## Results

### Patient selection

**Fig. 1:**
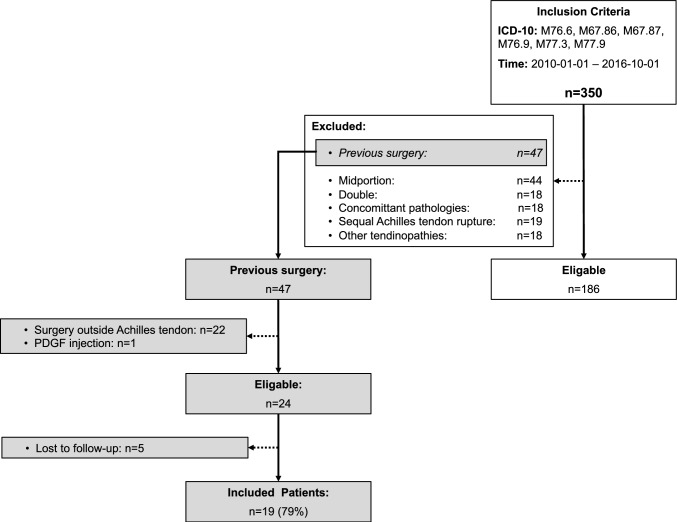
Flow-chart illustrating the patient selection for the “Revision IAT surgery”. *n* number of patients, *PDGF* platelet-derived growth factor

The overall patient selection is outlined in Fig. 1. Out of the 47 patients with revision surgery following failed IAT surgery, 24 were eligible, and 19 (79%) were included in the final analysis. The mean age of the patients was 50 ± 14 years, with 58% of the cohort being female. The left foot was affected in 32% of the patients.

### Patient rated outcome (FFI, VISA-A-G)

**Fig. 2:**
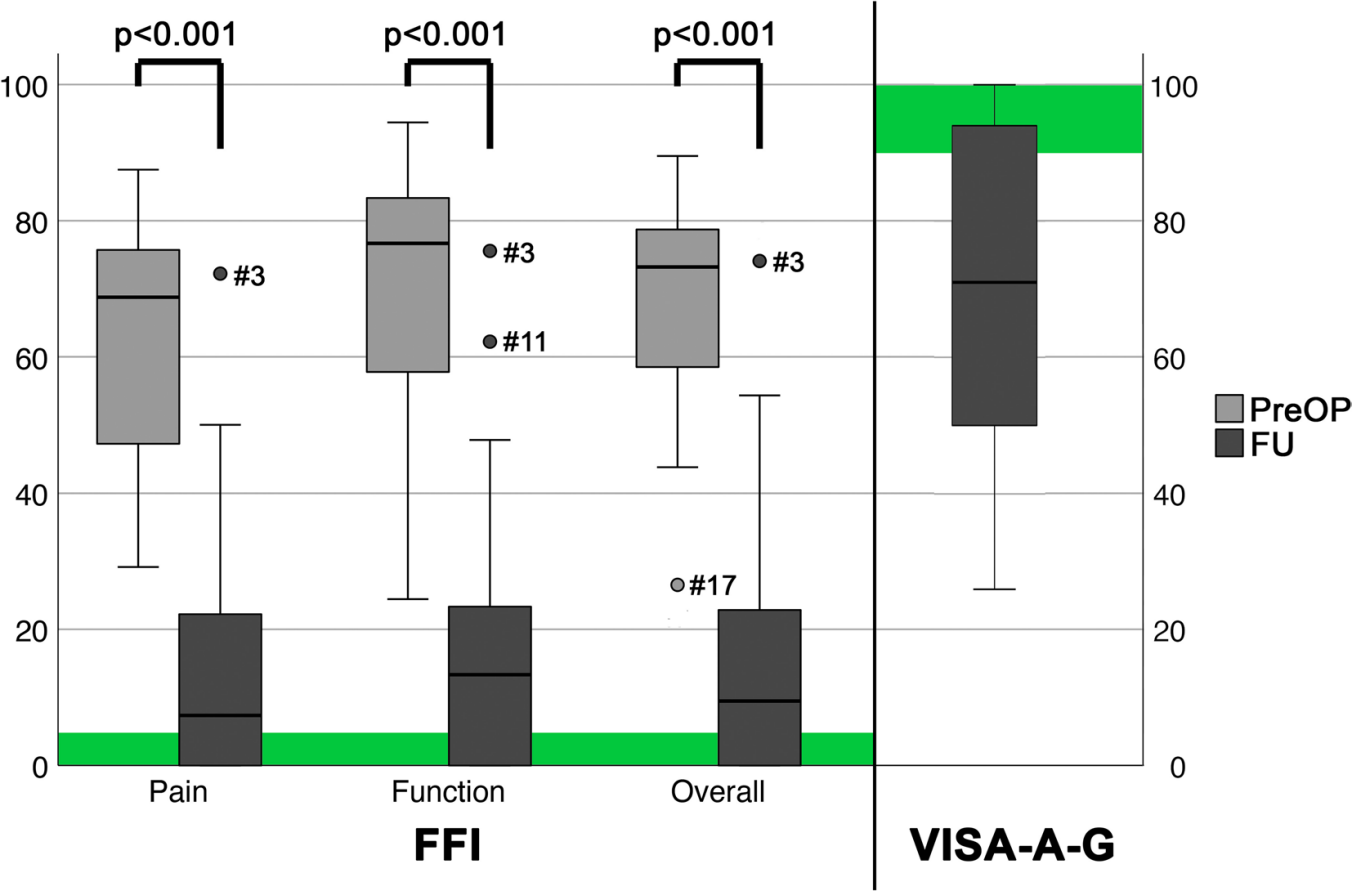
Patient rated outcomes. Boxplots compare preoperative and final follow-up FFI Scores and the VISA-A G at final follow-up for revision surgery of surgically treated IAT recurrence. Green boxes represent the score areas for healthy populations: FFI, ≤ 5 points; VISA-A-G, ≥ 90 points

The mean follow-up was 4.6 ± 2.2 years following the revision surgery. Figure 2 outlines the PROM results. The FFI Overall decreased from preoperative 68 ± 18 to 17 ± 22 points (< 0.001) at the final follow-up. A similar decrease was found for the FFI subscales Pain and Function. The VISA-A-G at the final follow-up was 70 ± 27 (Fig. 2). 36% of patients reached values comparable to a healthy reference population (≥ 90 points).

Per the postoperative FFI score, one patient (Fig. 2; #3) qualified as an outlier in all subscales. The patient was a 66-year-old female with an FFI Overall score of 74 and a VISA-A-G score of 66 points. The index procedure was performed 8 years ago. In the revision surgery, the postero-superior calcaneal prominence, a dorsal spur, and intratendinous calcifications were resected. A suture anchor reattached the Achilles tendon. She suffered a minor surgical site infection (SSI) which could be treated successfully non-operatively. At the final follow-up, she reported a persisting shoe conflict due to the scar. This patient was excluded from further analysis.

### Patient characteristics and surgical procedures

The patient characteristics and surgical procedures performed, excluding the outlier, are outlined in Table 1. Seven patients were treated by a MITA, in 11 patients the pre-existing para-achillary approach (4 × dorsomedial, 7 × dorsolateral) was extended as necessary. In total, 3.3 ± 1.1 surgical procedures were performed per patient. One surgical side infection occurred, and one patient suffered from a postoperative chronic regional pain syndrome. The surgical side infection was classified as minor complications and resolved with oral antibiotics.

**Table 1:** Overview of the overall cohort without the outlier (Patient #3) and group comparison between those patients with a good outcome (FFI ≤ 5) and impaired outcome (FFI > 5)

	Revision cohort n = 18	Good outcome FFI ≤ 5 (n = 7)	Impaired outcome FFI > 5 (n = 11)	p value
Age	49 ± 14	54 ± 15	46 ± 13	0.159
Sex (% female)	56%	43%	70%	0.350
BMI	27 ± 5	26 ± 4	29 ± 7	0.242
ASA	1.6 ± 0.7	1.7 ± 0.8	1.6 ± 0.7	0.793
Smoking (% smoker)	12%	14%	11%	1.0
DM (% DM)	12%	14%	11%	1.0
aHT (%aHT)	17%	29%	10%	0.537
Resection posterosuperior calcaneal prominence (Haglund’s exostosis)	83%	100%	70%	0.228
Resection dorsal spur	50%	57%	50%	1.0
Resection intratendinous calcifications	28%	14%	30%	0.603
Debridement Achilles tendon	83%	71%	90%	0.537
Detachment Achilles tendon > 50%	61%	86%	50%	0.304
Removal of previous suture anchor	17%	0%	30%	0.228
FHL transfer	6%	0%	10%	1.0
Mean number of surgical procedures	3.3 ± 1.1	3.3 ± 1.0	3.3 ± 1.3	0.979
FFI Overall—preOP	68 ± 19 (27–90)	63 ± 25	70 ± 15	0.265
FFI Overall—postOP	14 ± 17 (0–54; 41%)*	1 ± 2	22 ± 17	**0.002**
FFI Pain—preOP	64 ± 19 (29–88)	63 ± 22	64 ± 18	0.449
FFI Pain—postOP	12 ± 16 (0–50; 47%)*	1 ± 2	20 ± 17	**0.003**
FFI Function—preOP	70 ± 20 (25 ± 94)	64 ± 27	73 ± 16	0.210
FFI Function—postOP	15 ± 18 (0–62; 47%)*	1 ± 2	24 ± 18	**0.001**
VISA-A-G—postOP	71 ± 28 (26–100; 39%)*	95 ± 7	54 ± 19	**0.001**

*preOP* preoperative, *postOP* postoperative, *DM* diabetes mellitus, *aHT* arterial hypertension

*Percentage of patients with PROM values in the range of the average population (p < 0.004; Compared were the two patient groups FFI5 in bold)

### Factors affecting the patient-rated outcome

The group comparison did not reveal any significant influencing factors regarding demographics or surgical procedures (Table 1). As expected, also the FFI subscales Pain and Function, as well as the VISA-A-G, were significantly better in the average population group compared to the impaired patient group.

## Discussion

The study aimed to analyze the patient-rated outcome following revision surgery for surgically failed IAT. Revision surgery in recurrence cases showed a significant improvement for the FFI and VISA-A G after 4.6 ± 2.2 years. Still, 39% of patients reached FFI values comparable to a healthy reference population. No parameters could be identified to predict the outcome.

IAT is a common pathology in any foot and ankle practice. Various studies have assessed the lifetime risk, diagnostic measures, classifications, and conservative [26] and surgical therapeutic approaches [13, 27, 28]. Therefore, we have a rather clear road map on how to approach patients with a primary IAT. Still, recurrence following surgically treated IAT has been reported to range between 7 and 17% [18].

Recurrence following surgical treatment of IAT therefore appears to be a problem worth investigating. Still data on how to deal with IAT recurrence cases is scarce. The authors are actually not aware of any study reporting on conservative treatment of IAT recurrence. And for IAT revision surgery, the current study is only the second to report on the outcome. In the herein presented study, revision surgery resulted in FFI values comparable to a healthy population in 39% (FFI Overall 39%/Pain 44%/Function 44%). Although this is just half of that after primary surgery (FFI: 62%) [13], it still highlights the potential for revision surgery. The only other study was published in 2022 by Maffulli et al. [19]. They prospectively followed 33 patients undergoing revision surgery. At two-years follow-up, the average VISA-A score was 75 ± 29 points, which is in line with the herein observed 71 ± 28 (26–100) points after 4.6 ± 2.2 years. Knowing the expected patient rated outcome is of upmost importance in order to advice patients. This is of particular importance, as foot and ankle patients have been shown to have higher expectations of the postoperative outcome compared to the treating surgeon [29].

Although these data are promising, considering the fact, that they report on the outcome of revision cases, the two studies are not completely comparable. They first varied per the surgical approach. Whereas Maffulli et al. facilitated a Cincinnati approach [19], which has been reported to have less scarring [30]. The authors of the current study aimed predominantly for a MITA. Still, the decision on which approach to use in revision cases is not only determined by the pathologies that need to be addressed, but also by the index approach used for the index procedure. If a para-achillary or midline incision was used, the Cincinnati approach would run perpendicular to the initial approach, or vice versa. This might increase the risk of wound-healing complications. Still, it is comforting for surgeons that either approach can be used safely.

Secondly, the two studies varied per the inclusion criteria. Whereas the current study included all revision cases in their analysis, Maffulli et al. [19] excluded patients with calcifying IAT. In the current study, 58% of the patients had a calcified IAT (dorsal spur or intratendinous calcifications), all of which were addressed during revision surgery in all cases. Considering the comparable outcome of both studies, these differences might highlight the fact, that in revision surgery, surgeons should address all pathologies present. If addressed, even calcified IAT recurrence cases apparently respond well to surgery.

Despite its originality, the study presented herein has several limitations. First, it’s a rather small retrospective case-series. Still, the observed lost-to follow-up rate of 21% compares favourably to previous studies [10, 11, 31]. Second, the current study has no control group. One could argue that the invasiveness of the procedure itself is a risk factor for an impaired patient rated outcome. Less invasive alternatives could be endoscopic procedures [32] or dorsal closing wedge osteotomies [33], both of which have been proven effective in primary IAT cases. Third, no factors predictive for an impaired outcome could be identified in the current study. This again was most likely due to the limited sample size. Strengths of the current study are the well-defined patient collective, the detailed description of pathologies addressed, and the use of validated outcome scores.

## Conclusion

IAT revision surgery results in an improvement of the patients’ symptoms, but one-third of the patients recovered fully. This stayed true even in the case of a calcifying IAT. No factors could be identified predicting the outcome. Future studies should focus on the possible value of less invasive surgical procedures in revision cases.
